# Improving global integration of crop research: Field laboratories in realistic crop environments are needed

**DOI:** 10.1126/science.aam8559

**Published:** 2017-07-28

**Authors:** M. P. Reynolds, H. J. Braun, A. J. Cavalieri, S. Chapotin, W. J. Davies, P. Ellul, C. Feuillet, B. Govaerts, M. J. Kropff, H. Lucas, J. Nelson, W. Powell, E. Quilligan, M. W. Rosegrant, Ravi P. Singh, K. Sonder, H. Tang, S. Visscher, R. Wang

**Affiliations:** 1 International Maize and Wheat Improvement Center, Mexico D.F., Mexico; 2 Bill & Melinda Gates Foundation, Seattle, WA, USA; 3 U.S. Agency for International Development, Washington, DC, USA; 4 Global Plant Council and Lancaster University, Lancaster, UK; 5 CGIAR System Organization, Montpellier, France; 6 Crop Science Division, Bayer, Morrisville, USA; 7 Wheat Initiative and National Institute for Agricultural Research, Paris, France; 8 Scotland’s Rural College, Edinburgh, UK; 9 International Food Policy Research Institute, Washington, DC, USA; 10 Chinese Academy of Agricultural Sciences, Beijing, China; 11 Biotechnology and Biological Sciences Research Council, Swindon, UK; 12 Food and Agriculture Organization of the United Nations, Rome, Italy

**Figure:**
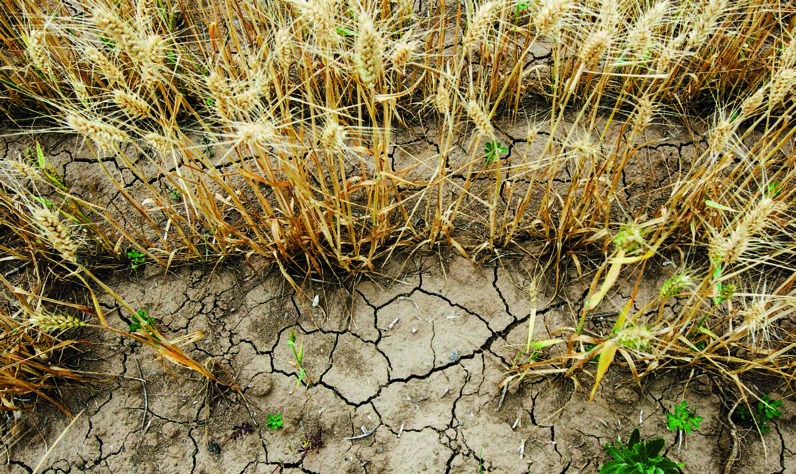


In recent decades, the scientific, development, and farm communities have contributed to substantial gains in crop productivity, including in many less developed countries (LDCs) (*1*), yet current yield trends and agri-food systems are inadequate to match projected demand (*2*). Addressing transnational crop challenges will require refinement of research infrastructure and better leverage of global expertise and technologies. Drawing on lessons learned from international collaboration in wheat, we outline how such a model could evolve into a Global Crop Improvement Network (GCIN) encompassing most staple food crops, providing access to wellcontrolled “field laboratories,” while harmonizing research practices and sharing data. Combined with socioeconomic and cropping systems research, a GCIN could revolutionize the ability to understand and model crop responses to environments globally and accelerate adoption of vital technologies.

Pioneering approaches for globally coordinated crop research (initially rice, wheat, and open-pollinated maize) emerged during the Green Revolution (*3*). The International Wheat Improvement Network (IWIN), part of the CGIAR system, tests new wheat genotypes at approximately 700 field sites in over 90 countries. Breeding, directed toward 12 different mega-environments that represent a range of temperature, moisture, and disease profiles, is conducted at strategic research hubs to develop around 1000 high-yielding, diseaseresistant lines. These are targeted to major agro-ecologies and are delivered annually as international public goods (IPGs). Data on adaptive responses of new lines is shared within IWIN to refine research and breeding methods (*4*–*6*). IWIN-related varieties cover more than half of the wheat area in LDCs, giving additional value (attributable to IWIN research) of between $2.2 and $3.1 billion per year, spread among resource-poor farmers and consumers (*6*). The benefit-cost ratio of this investment is over 100:1, without considering the added value of avoiding devastating disease pandemics by breeding for disease resistance (*5*), or the estimated 20+ million hectares of ecosystems that have been spared cultivation as a result of increased productivity (*7*). IWIN has amassed a database of over 20 million phenotypic data points that are beginning to be exploited, e.g., in modeling crop responses to climate changes (*4*).

**Transnational field testing could close gaps in crop yield. Severe drought in China has affected harvests**.

## ACHIEVING A GLOBAL NETWORK

Taking IWIN as a baseline, three developments are suggested for a GCIN-like approach to transnational field testing. First, expand the network to include other species, disciplines, and key actors in crop value chains while adopting and harmonizing best practices. Second, underpin parallel research in cropping systems investigating interactions between genotype, environment, and input management, to better understand and close yield gaps (*8*). Third, underpin national program capacity and develop research infrastructure at key agro-ecologies sites. Infrastructure such as high-throughput phenotyping (*9*) and remote-sensing platforms (*10*) has increased the scope and rigor of field-based research. By accessing a wider range of experimental factors (crops, environments, new technologies, input constraints, etc.) while facilitating sharing of research methods, tools, and data among a broader partner base, research is enhanced via regionally coordinated multilocation research. A systematic and better-equipped field network can bring more scientific rigor (as well as technologies) to the field, and reduce dependence of upstream work on environmentally controlled research facilities that are unrepresentative of cropping situations (*11*).

Shared research platforms may be the only equitable way to access sites that are analogs for future climate and disease threats. This is especially true for countries with a low range of environments (*12*). Many problems of global or regional concern can be tackled through a collective approach; for example, a public network of free-air CO _2_ enrichment facilities at key crop environments would provide guidance for climate adaptation. IWIN accesses key ecologies for global leverage, including screening for a highly virulent stemrust fungus (Ug*99*) in Kenya to avert a global pandemic (*5*), and the International Wheat Yield Partnership (IWYP), where outputs of basic research are translated into breeding.

Although the main role of a GCIN would be orchestrating field-based research across disciplines and environments, close links with national agricultural research services (NARS) could help the outputs and infrastructure of a GCIN to underpin downstream activities (*12*). These include innovative extension and decision support (radio, mobile phone, and other technologies) provided by nongovernmental organizations, private seed and input companies, and public extension programs. Such linkages would detect bottlenecks to adoption of new technologies.

## DATA INTEGRATION

Common standards are required if data are to be comparable across experimental variables, including germplasm, environments, and other research interventions. Highthroughput phenotyping and genotyping protocols have some agreed standards (*9*, *13*). Standardized protocols also add value in modeling studies where core data sets drive simulations, permitting alternative interventions to be evaluated and prioritized. Crop models have been used to estimate impacts of climate change on crop performance (*4*, *10*), yet many breeding and agronomy data sets do not fulfill core needs to drive models.

Research institutions and funding bodies could facilitate more timely data sharing by prioritizing publication of results linked to open-access data. Data sharing will drive standardization toward more precise descriptions of environments and experimental treatments, and more “searchable” databases (*14*). Although intellectual property (IP) rights are necessary incentives for private investment, greater access to data benefits all sectors. It would be mutually beneficial to carefully define “precompetitive” research so that private entities are encouraged to share nonsensitive data more routinely in precompetitive mode and when engaged in public-private partnerships (PPPs). Some transforming technologies could be made more accessible through nonexclusive licenses, while ensuring that industry received returns on investments.

## OVERCOMING BARRIERS

Networks like IWIN that have brought together a broad spectrum of partners—from the CGIAR system, academia, NARS, and the private sector—are now largely funded by competitive (as opposed to core) funds, with the attendant transaction costs and unpredictability of funding that limits a longerterm vision. Whereas the CGIAR has moved its core agenda in other directions (including upstream), many Western academic institutions are forging new bilateral projects with traditional CGIAR partners (i.e., NARS in LDCs). This reinforces the value of wellcoordinated multilateral partnerships to accelerate impacts, the kind of role a GCIN can provide. The expectation for crop research programs to deliver positive outcomes on the livelihoods of resource-poor people—another dimension of the CGIAR’s expanded remit (*15*)—is complex, as new crop technologies are just one part of an equation that includes factors such as credit, market forces, national policy, etc. New collaborative paradigms encompassing a broader range of stakeholders, as proposed here, would bolster such outcomes, e.g., in the context of microfinancing and other PPPs. Yet a 2015 international workshop on the promotion of PPPs organized by 10 international agencies—including the CGIAR—identified uncertainty in funding of international programs as a key risk for improved resilience of agri-food systems (*15*).

Increasingly high transaction costs associated with IPG work must also be addressed. These are partly driven by liability and IP issues, and partly by the prioritization of full accountability and risk minimization, which favor short-term, project-driven research over longer-term, multilateral research programs.

One way to initiate and finance a GCIN would be through structural re-arrangement within the CGIAR, whose annual budget is approximately US$900 million. For 2017, the total budget expected for crop research including trait discovery, variety development, and seed systems—is approximately $200 million, covering the major cereals, legumes, roots, and tubers. These crop networks would constitute the major components of a GCIN, while additional investment would underpin and improve NARS infrastructure, encompass underutilized crops—e.g., quinoa (*16*)— and achieve cohesion and a strategic vision. Some costs would be offset by economies of scale, including shared infrastructure, data, and best practices across crops. IWIN and other crop-testing networks rely heavily on voluntary data return, in exchange for shared germplasm and other technologies generated from initial investment in CGIAR programs. A GCIN would thus count on substantial leverage of a massive body of human, physical, and scientific capital from existing crop networks in return for shared benefits of multilateral collaboration and improved research infrastructure. Recently established crosscutting CGIAR platforms, including one to harness big data and another for modernization of breeding methods within CGIAR centers, would also contribute to a GCIN’s goals.

**“A successful GCIN would likely require a consortium of funding bodies…”**

Some multinational seed companies have invested heavily in infrastructure at key research locations, and the model is no less compelling for achieving food security through provision of IPGs (e.g., Feed the Future, CGIAR, Bill & Melinda Gates Foundation). Some elements of a GCIN could be supported by hybrid funding mechanisms such as the Foundation for Food and Agriculture Research model that requires 50% private industry investment to receive government funding, or the Phytobiome Alliance PPP. There is an IP challenge when private entities join forces, but many are moving in the direction of cooperating at the precompetitive level to share risks and costs associated with basic research. With clear definitions and agreements, investment by industry in precompetitive areas would allow private companies to develop products further down the line, while raising the technology bar for all. The notion that private entities would invest in a GCIN (with its LDC focus) is supported by two key facts. First, the G20 research priorities and those of LDCs served by the CGIAR often overlap, providing opportunities for international collaboration. Second, future markets for crop commodities will be dominated by current LDCs where population is growing fastest and diets are rapidly changing. The IWYP model is one example where IP arrangements involving global access to new germplasm have been agreed across a global PPP.

A successful GCIN would likely require a consortium of funding bodies to set the agenda and put governance in place according to their own criteria. An evaluation process should be designed to estimate ex ante and ex post returns to the network, and precedents are extremely favorable (*3*, *6*, *7*). At this stage, the best way to promote a GCIN is to elaborate a scientific rationale based on precedents and opportunities. ▀

## References

[1] Fischer R. A., Byerlee D., Edmeades G. O., Crop Yields and Food Security: Will Yield Increases Continue to Feed the World? ACIAR Monograph (Canberra, 2014).

[2] Ray D. K. et al., *PLOS ONE* 8, e66428 (2013). 23840465 10.1371/journal.pone.0066428 PMC3686737

[3] Pingali P. L., *Proc. Natl. Acad. Sci. U.S.A*. 109, 12302 (2012). 22826253 10.1073/pnas.0912953109 PMC3411969

[4] Gourdji S. M. et al., *Proc. R. Soc. B*. 280, 20122190 (2013). 10.1098/rspb.2012.2190 PMC3574297 23222442

[5] Singh R. P. et al., *Annu. Rev. Phytopathol*. 49, 465 (2011). 21568701 10.1146/annurev-phyto-072910-095423

[6] Lantican M. A. et al., Impacts of International Wheat Improvement Research, 1994-2014 (CIMMYT, Mexico, D.F, 2016).

[7] Stevenson J. R., Villoria N., Byerlee D., Kelley T., Maredia M., *Proc. Natl. Acad. Sci. U. S. A*. 110, 8363 (2013). 10.1073/pnas.1208065110 PMC3666715 23671086

[8] Lobell D. B., Cassman K. G., Field C. B., *Annu. Rev. Environ. Resour*. 34, 179(2009).

[9] Araus J. L., Cairns J. E., *Trends Plant Sci*. 19, 52 (2014). 24139902 10.1016/j.tplants.2013.09.008

[10] Lobell D. B. et al., *Nat. Clim. Chang*. 1, 42 (2011).

[11] Poorter H. et al., *New Phytol*. 212, 838 (2016). 27783423 10.1111/nph.14243

[12] Reynolds M. P., *J. Exp. Bot*. 63, 10 (2012). 10.1093/jxb/ers071 PMC3388823 22412185

[13] Pérez-Harguindeguy N. et al., *Aust. J. Bot*. 61, 167 (2013).

[14] Editorial, *Nat. Genet.* 47, 99 (2015).25627896

[15] Leeuwis C., Klerkx L., Schut M., *Glob. Food Security* 10.1016/j.gfs.2017.06.002 (2017).

[16] Ruiz K. B. et al., *Agron. Sustain. Dev*. 34, 349 (2014).

